# The effect of Schroth exercises added to the standard of care on the quality of life and muscle endurance in adolescents with idiopathic scoliosis—an assessor and statistician blinded randomized controlled trial: “SOSORT 2015 Award Winner”

**DOI:** 10.1186/s13013-015-0048-5

**Published:** 2015-09-18

**Authors:** Sanja Schreiber, Eric C. Parent, Elham Khodayari Moez, Douglas M. Hedden, Doug Hill, Marc J. Moreau, Edmond Lou, Elise M. Watkins, Sarah C. Southon

**Affiliations:** University of Alberta, Edmonton, Canada; University of Alberta, Alberta Health Services, Edmonton, Canada

**Keywords:** Adolescent idiopathic scoliosis, Schroth exercises, Randomized controlled trial, Quality of life, Muscle endurance

## Abstract

**Background:**

In North America, care recommendations for adolescents with small idiopathic scoliosis (AIS) curves include observation or bracing. Schroth scoliosis-specific exercises have demonstrated promising results on various outcomes in uncontrolled studies. This randomized controlled trial (RCT) aimed to determine the effect of Schroth exercises combined with the standard of care on quality-of-life (QOL) outcomes and back muscle endurance (BME) compared to standard of care alone in patients with AIS.

**Material and Methods:**

Fifty patients with AIS, aged 10–18 years, with curves 10–45 °, recruited from a scoliosis clinic were randomized to receive standard of care or supervised Schroth exercises plus standard of care for 6 months. Schroth exercises were taught over five sessions in the first two weeks. A daily home program was adjusted during weekly supervised sessions. The assessor and the statistician were blinded. Outcomes included the Biering-Sorensen (BME) test, Scoliosis Research Society (SRS-22r) and Spinal Appearance Questionnaires (SAQ) scores. Intention-to-treat (ITT) and per protocol (PP) linear mixed effects models were analyzed. Because ITT and PP analyses produced similar results, only ITT is reported.

**Results:**

After 3 months, BME in the Schroth group improved by 32.3 s, and in the control by 4.8 s. This 27.5 s difference in change between groups was statically significant (95 % CI 1.1 to 53.8 s, *p* = 0.04). From 3 to 6 months, the self-image improved in the Schroth group by 0.13 and deteriorated in the control by 0.17 (0.3, 95 % CI 0.01 to 0.59, *p* = 0.049). A difference between groups for the change in the SRS-22r pain score transformed to its power of four was observed from 3 to 6 months (85.3, 95 % CI 8.1 to 162.5, *p* = 0.03), where (SRS-22 pain score)^4^ increased by 65.3 in the Schroth and decreased by 20.0 in the control group. Covariates: age, self-efficacy, brace-wear, Schroth classification, and height had significant main effects on some outcomes. Baseline ceiling effects were high: SRS-22r (pain = 18.4 %, function = 28.6 %), and SAQ (prominence = 26.5 %, waist = 29.2 %, chest = 46.9 %, trunk shift = 12.2 % and shoulders = 18.4 %).

**Conclusions:**

Supervised Schroth exercises provided added benefit to the standard of care by improving SRS-22r pain, self-image scores and BME. Given the high prevalence of ceiling effects on SRS-22r and SAQ questionnaires’ domains, we hypothesize that in the AIS population receiving conservative treatments, different QOL questionnaires with adequate responsiveness are needed.

**Trial registration:**

Schroth Exercise Trial for Scoliosis NCT01610908.

**Electronic supplementary material:**

The online version of this article (doi:10.1186/s13013-015-0048-5) contains supplementary material, which is available to authorized users.

## Background

Adolescent idiopathic scoliosis (AIS) is the most common pediatric spinal deformity. It progresses most rapidly during the pubertal growth spurt. The prevalence in the general adolescent population has been reported to be up to 5.2 % [1] and the annual incidence about 2 % [2]. Rapid scoliosis progression leads to decreased self-esteem [3], mental health concerns [4], pain [5–8], respiratory complications [9] and limited function [6, 7]. These observations justify efforts to start the treatment early before the pubertal growth.

In North America the standard of care for scoliosis includes: observation for patients with curves between 10 and 25 °, and who are still growing; bracing for patients with curves between 25 and 45 ° during the growth phase, and spinal fusion for patients with curves >45 ° while the patients are still growing and with curves of >50 ° if the growth has ceased [10]. In contrast, European and recommendations from the Society on Scoliosis Orthopaedic and Rehabilitation Treatment (SOSORT) include scoliosis-specific exercises as a stand-alone therapy, as an add-on to braces, and during the post-surgical period [11–13]. Cost, culture [14], social standards [15] or, possibly, differing appraisals of the quality of research involving exercise treatments [10] could explain these differences in treatment recommendations.

Standard care consisting of observation, bracing and surgery has its shortcomings. Patients with curves ≤25 ° are routinely radiographed to assess the progression every 4–6 months, but no treatment is offered. Over 6 months, scoliosis curves are expected to progress 5.4 ° on average, with some fast progressive curves predicted to increase by as much as 9.6 ° [16]. With quick curve progression, body shape asymmetries also develop affecting the trunk, pelvis, ribs, shoulders, lumbar and waist areas. Symptoms such as pain, psychological issues, and a decreased quality of life (QOL) are common. Moreover, the likelihood of experiencing such symptoms during adulthood can increase during observation [17–19]. Brace-wear may induce stress, negatively affect self-esteem [20], produce soreness, discomfort with activity, torn clothing, as well as, limitations in sport, physical activity and social events [20, 21]. Although, brace treatment alters the natural history and limits progression during the growth phase [22], patients treated with brace and observation, when analyzed without regards to the curve size at the end of growth, have been observed in the long term to have similar rate of surgical intervention and curve progression after maturity [23]. While surgery reduces deformity and prevents further curve progression [19], in the long term, surgically treated patients have more degenerative disc changes than controls, more frequent lumbar or bodily pain, reduced physical functioning and general health as well as more sick-leaves due to back pain [7].

Generally, exercises are well received by patients [24]. Patients and parents frequently express interest toward exercises [15]. Tones et al. suggest that persons with scoliosis who exercise regularly, show higher self-esteem and have better psychological outcomes [21]. This strengthens the importance of physical therapy as a treatment alternative for AIS.

Several systematic reviews on the effects of exercises for scoliosis have reported promising results [25–29]. Fusco et al’s systematic review [29] concluded that physical exercises slowed the progression of scoliosis and/or reduced curve severity measured by the Cobb angle, improved neuromotor control [30], respiratory function [31], back muscle strength [31], and cosmetic appearance [31, 32]. However, QOL outcomes were not routinely assessed.

Since Fusco’s review, three studies on exercises were published. Noh et al’s prospective controlled study of 32 patients demonstrated that the scoliosis-specific “3D corrective spinal technique” was superior compared to the conventional exercises in improving radiological and SRS-22 questionnaire outcomes [33]. However, the conventional exercise group also experienced significant improvement of QOL reinforcing the evidence suggesting that various types of exercise can benefit QOL of patients with AIS [21]. In a recent RCT, Monticone et al. found that scoliosis-specific active self-correction and task-oriented exercises, consistent with Scientific Exercise Approach for Scoliosis (SEAS) [34] significantly improved the Cobb angles and the QOL measured at skeletal maturity by the SRS-22r questionnaire (by 0.75 to 0.89/5) while the traditional spinal exercises were associated with stable outcomes [34]. The study included 110 skeletally immature patients with AIS and curves <25 ° at baseline. In a third recent RCT, preoperative aerobic training improved the QOL in 40 surgical candidates with AIS [35].

Among all scoliosis-specific exercise approaches, the Schroth method is among the most studied and widely used. The Schroth method consists of scoliosis-specific sensorimotor, postural and breathing exercises [36]. Auto-correction defined as the patient’s ability to reduce the spinal deformity through active postural realignment of the spine in three dimensions [29] is a fundamental component of the Schroth method. Auto-correction is achieved through self-elongation and postural corrections that are specific for each curve pattern, and is eventually integrated in daily activities. In several cohort studies, the Schroth method demonstrated positive outcomes on back muscle strength [31], breathing function [31], slowing curve progression [37], improving Cobb angles [31, 37] and decreasing the prevalence of surgery [38].

The results from a large case–control study [39] suggest that the back muscle endurance of scoliosis patients is significantly lower than in people without scoliosis. Paraspinal muscles are needed to maintain spinal alignment throughout the day. To our knowledge this outcome has not been investigated in a Schroth exercise study.

To our knowledge no RCT or prospective controlled studies have been conducted on the effect of Schroth exercises. Moreover, most exercise studies did not blind the assessors, report on compliance, intention-to-treat analyses, or on recruitment strategies. The promising effect of Schroth exercises on QOL should be confirmed in a RCT conducted by independent researchers, not involved in the development and promotion of or profiting from the approach, to limit investigator bias, and to address the methodological limitations of prior studies.

Therefore, the objective of this RCT on Schroth exercises was to determine the effect of a 6-month Schroth exercise intervention in conjunction with standard of care (observation and bracing) on QOL, perceived appearance and back muscle endurance, compared to the standard of care alone in patients with AIS.

## Methods

### Study design

This was a phase II assessor- and statistician-blinded, randomized controlled clinical trial. The full protocol for this study has been published [40]. The CONSORT flow chart for this RCT has been reported in Additional file 1.

### Participants and therapists

We consecutively enrolled patients with AIS from the scoliosis clinic at our institution. Inclusion criteria were: 10–18 years old, both genders, curves 10–45 °, Risser 0–5 (all skeletal maturities) and ability to travel to weekly visits. Exclusion criteria were: diagnosis other than AIS, planning surgery, having had surgery, previously weaned from brace, being scheduled for clinical follow-up later than in 6 ± 2 months or being discharged from the clinic when approached to participate. We obtained assent from the patients and informed parental consent prior to the enrolment. This study was approved by the University of Alberta Health Research Ethics Board (Pro00011552).

The main Schroth-certified therapist had 3 years of Schroth therapy experience and provided 95 % of the therapy sessions. Another certified therapist filled in as needed.

### Randomization and masking

A research coordinator invited eligible patients attending regular scoliosis clinic visits to participate in the study. Within 2 weeks from the visit, a researcher contacted interested patients to obtain consent and book a baseline evaluation. After an initial exam confirming eligibility and collecting baseline data, participants were randomized using a computer-generated sequence in pre-sealed envelopes into the Schroth exercises or the control group. We used random size (4–8) blocked randomization stratified for the four Schroth curve types [41] to ensure allocation of a balanced number of participants in both arms of the study (25 per group) for each curve type.

Therapists and patients could not be blinded when offering or receiving the Schroth treatment. However, participants were asked not to reveal their group allocation to ensure blinding of the evaluator. The statistician was not aware of the data coding.

### Intervention

#### Schroth exercises added to standard care (experimental) group

The 6-months supervised Schroth exercise intervention included five initial 1-h long private training sessions delivered during the first two weeks after baseline, followed by weekly 1-h long group classes combined with a 30–45 min daily home exercise program. Exercises are presented in Additional file 2 with a description of the corrective movements required, the curve type for which they are recommended, the level of passive support involved, whether they offer a static or dynamic challenge and the dosages recommended. A Schroth curve type classification algorithm [41] and algorithms to guide the exercise prescription and progression for each of the four Schroth curve types [42] were developed for this trial to standardize exercise delivery [40]. Participants in the experimental group also received bracing if the SRS bracing criteria [43] were met.

Compliance was monitored using exercise logbooks, verified daily by a parent and weekly by a therapist. During each class, adequate exercise performance was assessed using a checklist. To maximize compliance, we provided home equipment, access to facilities, and promoted parental involvement. When compliance dropped below 70 %, we tried to resolve the issues cooperatively with patients and parents [44, 45]. Attendance was calculated as a percentage of the prescribed visits attended, and compliance as a percentage of the prescribed home exercise dose completed over the course of the 6 months of treatment.

#### Standard care (control) group

Control subjects received the standard of care, consisting of observation or bracing if the SRS bracing criteria [43] were met. The SOSORT Management for bracing guidelines checklist is presented in Additional file 3 demonstrating that all but 3 of 44 criteria were met [46].

Controls attended study assessments, but not the therapy sessions.

### Measurements

The outcomes collected at baseline, three and 6 months included: Biering-Sorensen back muscle endurance, Scoliosis Research Society (SRS-22r) and Spinal Appearance Questionnaire (SAQ) scores.

The *Biering-Sorensen test* is a validated test assessing the isometric endurance of the trunk extensor muscles. This test measures the duration (in seconds) a subject is able to hold the trunk in extension while fixed to a table. The test is stopped when a subject can no longer control the posture or when 240 s have been reached [47]. The test-retest reliability was shown to be adequate ICC = 0.85(CI 0.76–0.90) with a standard error of measurement (SEM) of 15.6 s [48]. The test was validated for measuring back muscle fatigue [49].

The SRS-22r questionnaire is a scoliosis-related QOL questionnaire that assesses five domains: function, pain, self-image, mental health (five questions each), and satisfaction with care (two questions) [50]. Each question is scored from 1 to 5, where 1 is the worst, and 5 the best. The SRS-22r has adequate test-retest reliability and validity [51]. We analyzed the total score, function, pain and self-image domains, because those outcomes are deemed the top priority in conservative treatment for scoliosis [52].

The SAQ measures changes in patients’ perception of their deformity using 20 questions including standardized drawings. It assesses the following domain scores: trunk shift, waist, kyphosis, prominence, chest, shoulders, general and curve [53]. Each item is scored from 1 to 5, where 1 is the best, and 5 the worst. In surgically treated patients, the SAQ is responsive with adequate psychometric properties (test-retest reliability of 0.57 to 0.99 for the different scale items and a Cronbach alpha of 0.7) [53]. For the purposes of our study, we considered all but the SAQ kyphosis domain because this study did not focus on kyphosis corrections.

Self-efficacy questionnaire (SEQ) scores were collected at baseline as a covariate for the analysis. This validated questionnaire measures the belief in one’s own ability to complete a task (defined as corrective exercises) successfully using eight items rated from one (Disagree a lot) to five (Agree a lot) [54, 55]. Physical activity levels in adolescent girls are related to self-efficacy beliefs [56, 57]. Self-efficacy was found to be a moderator of the relationship between declines in physical activity and perceived social support [56].

### Statistical analysis

Descriptive statistics were calculated for baseline demographics and radiographs, for the entire sample, and for the patients who dropped out.

For the continuous outcomes, to assess differences in group changes from baseline to 3 to 6 months while adjusting for important covariates, we used linear mixed effects models analysis. For the ordinal outcome, SAQ curve, which is based only on one item with five levels, we used generalized linear mixed effects model analysis. Separate analyses were conducted for each outcome to assure the best covariate set was selected in the model. Covariates considered included age, weight, height, self-efficacy, whether a person wore a brace or not, and Schroth scoliosis classification. For covariates selection, we used the stepwise selection method using the Akaike information criterion (AIC) [58]. Outcome variables were transformed as needed to ensure meeting the normality assumptions. All final models included group, time and their interaction even if they were not retained by the stepwise selection methods. Time was coded into two covariates—Time2 and Time3, where Time3 denoted an effect over time from baseline to 3-month, and Time2 the effect from 3 to 6-month follow-up.

Both ITT and PP analysis were performed. ITT analysis included all subjects as they were randomized regardless of whether they completed the intervention as randomized, used co-interventions, their compliance with the treatment, or whether they dropped out [59]. ITT analysis was performed using the linear interpolation method [60], in which values immediately surrounding the missing data are joint by a line. The line joining the first and the last non-missing value, which represents the average progression of the actual individual trajectory is considered.

Statistical analyses for the continuous variables were performed using the statistical program R [61]. For the ordinal outcome we used the GLIMMIX procedure within the SAS program.

### Sample size justification

To detect a 0.50 effect size when comparing the change in the primary outcome between two groups with 80 % power using a two-tailed 0.05 hypothesis test, and considering a 0.6 correlation between repeated measures, 50 patients per group were needed [62]. However, the study ended after recruiting 50 participants when funding was received to continue the study as a multicenter RCT with slightly different participants’ criteria (Trial registration NCT01610908).

## Results

Fifty eligible and consenting patients were recruited. Treatment groups did not differ at baseline for gender, age, height and Risser sign (Table 1). Controls had higher mean weight by 4.4 kg than the experimental group. The mean age was 13.4 years (SD = 1.6). The mean largest curve was 28.5 ° (SD = 8.8 °), and the mean Risser was 1.60.

**Table 1:** Baseline characteristics within each group

	Schroth exercises + Standard of care (95 % Confidence interval), *N* = 25	Standard of care (95 % Confidence interval), *N* = 25
Age (years)	13.5 (12.7 to 14.2)	13.3 (12.7 to 13.9)
Girls	23	24
Braced	17	17
Height (m)	1.60 (1.6 to 1.6)	1.60 (1.6 to 1.6)
Weight (kg)	45.9 (42.6 to 49.1)	50.5 (47.1 to 54.0)
Largest curve (°)	29.1 (25.4 to 32.8)	27.9 (24.3 to 31.5)
Sum of curves (°)	48.1 (39.1 to 57.2)	54.3 (44.9 to 63.6)
Risser sign (0 to 5)	1.76 (1.10 to 2.45)	1.44 (0.77 to 2.11)
Risk of progression [79] (%)	65	65
SRS-22r Function (1 to 5)	4.60 (4.46 to 4.74)	4.55 (4.38 to 4.72)
SRS-22r Pain (1 to 5)	4.46 (4.26 to 4.67)	4.19 (3.92 to 4.47)
SRS-22r Self Image (1 to 5)	3.91 (3.65 to 4.17)	3.82 (3.55 to 4.08)
SRS-22r Total (1 to 5)	4.25 (4.09 to 4.40)	4.14 (3.96 to 4.31)
SAQ General (1 to 5)	2.92 (2.55 to 3.29)	2.89 (2.49 to 3.28)
SAQ Curve (1 to 5)	2.16 (1.96 to 2.36)	2.21 (2.03 to 2.38)
SAQ Prominence (1 to 5)	1.64 (1.41 to 1.87)	1.71 (1.48 to 1.93)
SAQ Trunk shift (1 to 5)	1.90 (1.68 to 2.11)	1.90 (1.68 to 2.11)
SAQ Waist (1 to 5)	2.75 (2.04 to 3.44)	2.64 (1.94 to 3.33)
SAQ Shoulders (1 to 5)	2.48 (2.04 to 2.92)	2.56 (2.16 to 2.96)
SAQ Chest (1 to 5)	2.14 (1.49 to 2.79)	2.19 (1.60 to 2.78)
Biering-Sorensen test (sec)	109.60 (87.67 to 131.53)	112.32 (85.47 to 139.17)

The number of patients wearing braces was even among groups. The type of braces worn by the patients, as well as the prescribed dosage was well balanced between the groups. There were 17 patients per group who wore a brace. Braces prescribed to the patients included: thoraco-lumbo-sacral orthosis (also known as TLSO and Boston brace) (*N* = 19), Providence (*N* = 2) and Charleston (*N* = 11) braces. Two patients wore a combination of Providence/TLSO braces. Of 34 patients wearing a brace, 13 wore theirs at night time only, 17 full time (20–23 h), one while at home, one 7 h, one 10–15 h, and one 14 h per day. The types of braces worn by the patients were balanced between groups. In the Schroth group six patients wore Charleston, nine TLSO and two wore a combination of Providence/TLSO braces. In the control group five patients wore Charleston, 10 TLSO and two Providence braces. The prescribed dosage was also balanced (7/night, 9/full time and 1/while at home in the Schroth group, and 6/night, 8/full time, 1/14 h, 1/10–15 h and 1/7 h in the control group).

Curve types based on the Schroth classification were as follows: 3c (*n* = 7) with a thoracic dominant deformity and balanced pelvis, 3cp (*n* = 15) with thoracic dominant deformity and pelvic shift toward the thoracic concave side, 4c (*n* = 5) with double major curves and balanced pelvis, and 4cp (*n* = 23) with thoracolumbar/lumbar dominant deformity and the pelvic shift toward the lumbar concave side. The number of patients within each classification was balanced between groups with no more than one subject difference for a given curve type.

Interestingly, the Schroth group had a bit better baseline SRS-22r questionnaire scores, ranging from 0.05 for function to 0.27 for pain. Despite those differences the confidence intervals between the groups were clearly overlapping. SAQ questionnaire scores were more balanced among the groups. Schroth group had slightly worse hold time on the Biering-Sorensen back muscle endurance test, but again with clearly overlapping confidence intervals.

Baseline, 3 and 6 months adjusted mean estimates from the ITT analysis and associated significance values for the outcomes based on the linear mixed effects models are presented in Table 2. Detailed significance and model coefficient results from the ITT and PP analyses are presented in Additional file 4.

**Table 2:** Adjusted mean estimates, standard errors and associated significance values for the SRS-22r, Spinal Appearance Questionnaire scores and the Biering-Sorensen test by visit and group predicted by the linear mixed effects models for the Intention-To-Treat analysis (ITT)

	Schroth (*N* = 25)		Control (*N* = 25)		0 to 3 months difference in change between groups	3 to 6 months difference in change between groups
	Mean	SE	Mean	SE	*p-*value	*p-*value
(SRS 22r Pain baseline)^4^	422.84	31.53	348.63	31.36	0.45	/
(SRS 22r Pain 3 month)^4^	460.76	32.37	415.71	32.62		0.02*
(SRS 22r Pain 6 month)^4^	525.99	33.38	395.68	32.36	/	
SRS 22r Self-Image baseline	4.00	0.12	3.91	0.12	0.14	/
SRS 22r Self-Image 3 month	3.85	0.12	3.97	0.13		<0.05*
SRS 22r Self-Image 6 month	3.98	0.13	3.81	0.13	/	
(SRS 22r Function baseline)^4^	444.54	46.83	413.56	49.34	0.67	/
(SRS 22r Function 3 month)^4^	442.84	47.44	395.94	50.45		0.43
(SRS 22r Function 6 month)^4^	485.29	48.48	408.33	50.68	/	
SRS 22r Total baseline	4.25	0.07	4.15	0.07	0.83	/
SRS 22r Total 3 month	4.29	0.07	4.18	0.07		0.08
SRS 22r Total 6 month	4.40	0.07	4.15	0.07	/	
$\sqrt{SAQ\ prominence}$ baseline	1.13	0.08	1.16	0.08	0.06	/
$\sqrt{SAQ\ prominence}$ 3 month	1.26	0.08	1.16	0.08		0.33
$\sqrt{SAQ\ prominence}$ 6 month	1.26	0.08	1.22	0.09	/	
SAQ Shoulders baseline	2.48	0.20	2.57	0.20	0.38	/
SAQ Shoulders 3 month	2.74	0.19	2.51	0.18		0.21
SAQ Shoulders 6 month	2.70	0.20	2.70	0.19	/	
(SAQ Waist^^-0.3^) baseline	0.86	0.03	0.87	0.03	0.46	/
(SAQ Waist^^-0.3^) 3 month	0.84	0.03	0.87	0.03		0.58
(SAQ Waist^^-0.3^) 6 month	0.81	0.03	0.87	0.04	/	
SAQ Trunk shift baseline	1.75	0.19	1.74	0.20	0.30	/
SAQ Trunk shift 3 month	1.93	0.19	1.69	0.20		0.26
SAQ Trunk shift 6 month	1.92	0.19	1.94	0.20	/	
log (SAQ Chest baseline)	0.39	0.25	0.49	0.25	0.99	/
log (SAQ Chest 3 month)	0.58	0.25	0.68	0.25		0.13
log (SAQ Chest 6 month)	0.74	0.28	0.62	0.28	/	
SAQ General baseline	2.71	0.18	2.64	0.18	0.71	/
SAQ General 3 month	2.84	0.19	2.44	0.19		0.60
SAQ General 6 month	2.64	0.21	2.41	0.20	/	
Sorensen baseline	117.38	12.19	120.96	12.30	0.04*	/
Sorensen 3 month	149.63	12.40	125.77	12.97		0.89
Sorensen 6 month	154.10	12.58	132.09	12.83	/	

All significant differences at the level of <0.05 are marked with*

### Dropouts

Only six of 50 randomized patients dropped out (12 %): four in the Schroth and two in the control group. Of those, there were four girls (one in the control and three in the Schroth group) and two boys (one per group). Dropouts had smaller largest curve (mean Cobb 23 °, SD = 5.27) than the entire sample (Table 1).

### Compliance

Patients who completed the study had high compliance: 85 % of visits were attended and 82.5 % of the home exercise program was completed. Including the dropouts, the compliance was 76 % of visits attended and 73 % of the prescribed home program exercise completed.

### Results for outcomes with significant differences between groups

#### SRS-22r pain

SRS-22r pain was transformed to its power of four—(SRS-22r pain)^4^ (Additional file 4). The change in the transformed pain score from baseline to 3-months did not differ between groups (−29.16, *p =* 0.45) (Table 2). However, from 3 to 6 months, the Schroth group experienced significant improvement compared to the control group (85.25, *p =* 0.03). Covariates included Age and SEQ, but did not have a significant main effect on pain.

#### SRS-22r self-image

From baseline to 3 months, self-image decreased in the Schroth and improved in the control group, but this −0.22 difference was not significant (*p =* 0.14) (Additional file 4 and Table 2). However, from 3 to 6 months, the self-image in the Schroth group improved, while it deteriorated in the control group and the difference was significant (0.30, *p =* 0.049). Only brace-wear was retained as a covariate but it did not have a significant main effect.

#### Biering-Sorensen test

After 3-month of follow-up, the Schroth group had significantly longer hold time than the controls (32.3 s vs. 4.8 s, *p* = 0.04) when controlling for age, self-efficacy and brace wear (Additional file 4 and Table 2). The change in the hold time from 3 to 6-months treatment was not significant between groups (−1.86, *p =* 0.89). None of the covariates had a significant main effect.

### Other questionnaire scores

There were no other statistically significant differences between groups for the change in the remaining questionnaires scores (Table 2 and Additional file 4).

### Reports of the significant main effects of covariates on outcomes in both groups

#### SRS-22r function

To meet normality assumption, SRS-22 function was transformed to its power of four -(SRS-22r function)^4^. Covariates included weight and curve classifications, with only curve classification having a significant main effect. The best function score was observed for the 3c curve type. The differences in the function domain between patients classified as 3c vs. 3cp, 4c or 4cp were all statistically significant (*p* = 0.01, *p* = 0.04, and *p* = 0.02, respectively). The 3cp, 4c and 4cp curve types did not differ significantly in function (Table 2 and Additional file 4).

#### SRS-22r total

The covariates retained included height, SEQ and age, but only age had a significant main effect, such that for every 1-year increase in age, the SRS-22r total dropped by 0.08 (*p =* 0.047) (Table 2 and Additional file 4).

#### SAQ prominence

To meet the normality assumption, the SAQ prominence was transformed to its square root. Only classification was retained as covariate, with 3cp having a significant main effect. Best scores were observed in patients classified as 3c. Those with a 3cp curve type had the worst scores of all and were the only type significantly worse than the 3c classification (0.25, *p =* 0.00) (Table 2 and Additional file 4).

#### SAQ waist

SAQ waist was transformed to its power of −0.3 to meet the normality assumption—(SAQ waist)^-0.3^. The model retained self-efficacy and brace-wear covariates. Self-efficacy was divided into covariates SEQ (overall effect of SEQ) and SEQ2 (the effect of SEQ scores when ≥35) (Table 2 and Additional file 4).

The patients who scored ≥35 of the SEQ had significantly worse SAQ waist scores than the ones with lower self-efficacy (0.10, *p* = 0.01). Likewise, patients who wore brace had worse waist scores than those without (0.08, *p =* 0.03).

#### SAQ trunk shift

Age, height and curve type were retained as covariates. Patients aged 10 and 11 behaved differently. To address this difference, age was divided into covariates Age and Age 10–11, but only Age 10–11 had a significant main effect. Ten and 11 years old patients had better scores on average by 0.83 points than their older counterparts (*p* = 0.00). Taller patients also had better scores (−1.89, *p* = 0.02) where for every 1 cm increase in height, patients had better score by 0.02. Patients with 3c curve types had significantly better score compared to patients with 3cp and 4cp curve patterns (by 0.49, *p* = 0.01 and 0.36, *p* = 0.047, respectively) (Table 2 and Additional file 4).

#### SAQ general

Height and brace wear were retained as covariates. Patients who wore a brace had significantly better scores by 0.72 (*p* = 0.00) (Table 2 and Additional file 4).

#### SAQ curve

Covariates included brace wear and classification. The model predicted that persons classified into 3cp have about 90 % higher odds of having score of >3, indicating worse outcomes (*p =* 0.01) (Table 2 and Additional file 4).

## Discussion

This is the first RCT investigating the effect of Schroth exercises on SRS-22r, SAQ questionnaires’ scores and back muscle endurance. Schroth exercises added to standard of care improved the SRS-22r pain scores and back muscle endurance after 3 months, and the self-image scores after 6 months of intervention. The Schroth intervention did not have significant effect on other outcomes.

In the only prospective study on Schroth exercises that examined the back muscle properties, strength, rather than endurance, was assessed using manual muscle testing scores ranging from 1 to 5 [31]. Otman et al. found that muscle strength increased significantly after 1 year. In two other studies, supervised resistive rotational exercises significantly increased strength after 4 months [30, 63]. To our knowledge, for the first time, Schroth exercises combined with the standard of care have been demonstrated to increase the back extension endurance.

Only one other study retrospectively investigating Schroth exercises and spinal stabilization compared to stabilization alone tested their effect on the SRS-22r, but not the SAQ questionnaire [33]. Noh et al. reported better SRS-22r results at 4 months for both groups but the experimental group demonstrated greater benefits, but significantly only for self-image (from 3.3 ± 1.2 to 4.2 ± 1.0) and the total score (from 3.8 ± 1.8 to 4.5 ± 0.4). Monticone et al’s recent RCT found positive effects of scoliosis-specific active self-correction and task-oriented exercises on changes on the Cobb angles and SRS-22r scores at skeletal maturity in 110 patients with AIS and curves <25 ° compared to standard rehabilitation exercises [34]. The authors reported more than 0.75 points improvement on all SRS-22r domains in the experimental group. In the present study, Schroth exercises improved pain and image scores at different follow-ups, but the change was in the range from 0.13 (image) to 0.17 (pain) points.

The larger effect on QOL observed in these studies may be due to the higher frequency of visits in Noh et al’s trial (2–3/week vs. 1/week in the present study) [33] and to the larger duration of Monticone’s trial (until maturity vs. 6 months in our study) [34]. Conversely, patients examined in these studies [34 had smaller curves than our sample but, interestingly, their scores on the SRS-22r questionnaire’s domains at baseline were worse and comparable to scores observed in surgical candidates leaving more room for measuring improvements on the questionnaires [64].

In the present study, the baseline values on the SRS-22r and SAQ questionnaires’ domains demonstrated high ceiling effects: SRS-22r (pain = 18.4 %, function = 28.6 %), and SAQ (prominence = 26.5 %, waist = 29.2 %, chest = 46.9 %, trunk shift = 12.2 % and shoulders = 18.4 %). The percentage of patients who scored ≥4 on the SRS-22r for which the best score is 5 and ≤2 on the SAQ domains where the best scores is 1 was also high: SRS-22 (total = 71.3 %, image = 47 %, pain = 77.5 % and function = 100 %) and SAQ (general = 18.3 %, curve = 79.6 %, prominence = 89.7 %, waist = 48 %, chest = 65.3 %, trunk shift = 77.5 % and shoulders = 38.8 %). High scores possibly limited the ability of these questionnaires to measure larger improvements. This finding is consistent with the results of a recent study that investigated the responsiveness of the SRS-22r questionnaire in patients with AIS treated with braces and exercises [65].

Other studies also demonstrated a high prevalence of ceiling effects in patients with smaller curves treated conservatively on the SRS-22r [66] and the SAQ [67]. Patients with AIS with curves <45 ° normally are in good health, and have a high level of function [68, 69]. Moreover, the SRS-22r and the SAQ questionnaires were originally designed for the surgically treated patients with AIS, who generally experience more scoliosis-related adverse symptoms that affect their QOL to a greater extent.

Recently, Caronni et al. performed Rasch analysis of the Italian version of the SRS-22 in patients tested before undergoing any form of treatment with curves ranging from low curve severity to pre-surgical [70]. They also found the SRS-22r to be limited by high ceiling effect. The authors suggested using seven items out of 22 using the Rasch analysis with adjusted scoring to create a more responsive questionnaire, the SRS-7. Despite the SRS-7 questionnaire displaying adequate Rasch characteristics for detecting change over time on a continuous scale, to our knowledge, the SRS-7 has not yet been used to measure the effect of treatments. Further, because of the small number of items and the narrow QOL range expressed by participants, the SRS-7 reliability is low (0.63) and only two QOL strata could theoretically be distinguished (high and low QOL) possibly raising doubt about its responsiveness [70].

To our knowledge, no alternative validated and more responsive questionnaires capable of capturing improvements in patients with AIS curves <45 ° and treated conservatively exist. Parent et al. [71], studied the Scoliosis Quality of Life Index which had been developed for use in younger children and found even more problems with ceiling effects than with the SRS-22r. Different tools, such as patient self-reported Trunk Appearance Perception Scale (TAPS) [72] or a clinician-reported Trunk Aesthetic Clinical Evaluation (TRACE) [73] might be more responsive in conservatively treated patients with AIS. However they have not been routinely used. The development of a new tool specific for this group of patients with AIS may be required to monitor quality of life and perceived appearance.

### On effect of some covariates

#### Classification

Some covariates had significant effects on outcomes in both groups. Classification was an important covariate for the SAQ trunk shift, waist, prominence, curve and SRS-22r function. For all these scores, the 3c curves had the best scores overall compared to other patients. In our sample, patients classified into the 3c group had the smallest curve magnitude, which could explain their highest scores compared to the other patients. Patients with the 3cp curve types were more likely to have worse curve and function scores. The 3cp curve pattern characterizes a more asymmetric posture. In our sample, patients with the 3cp classification had largest curves, which might explain the propensity toward worse scores on these outcomes.

#### Brace wear

Wearing a brace was associated with SAQ waist and general scores, such that the patients who wore brace had worse waist, but better general scores. In contrast to the waist domain, the SAQ general score are based on items with no reference to a specific anatomical body part, but rather represent a patient’s general appearance expectations, which could explain the opposite direction of the association of brace with these two domains. Having better scores in braced patients is not in agreement with previous studies, which suggests that braced patients have significantly more distorted perceived body appearance compared to the patients who do not wear a brace and have similar curve magnitudes [71, 74]. This difference may be because we report short-term (6 months of treatment) rather than long-term results (16 years after maturity).

In this study, finding that wearing a brace influenced our models for some outcomes does not mean that bracing has a therapeutic effect on the outcomes over time. Merely, this alerts us to the fact that patients meeting criteria for wearing a brace and wearing one during the study present SAQ scores that differ from patients not wearing a brace. This observation applies to patients braced in both the control and the Schroth groups. The present trial did not randomize patients meeting criteria to be prescribed a brace to a no-brace group, which would be required to conclude about the effectiveness of bracing.

#### Age

The 10–11 years olds had better SAQ waist scores than others and older patients had worse SRS-22r total score. We also found that older patients had worse SRS-22 total score. Younger patients might not be yet sensitized to the perception of their posture or scoliosis signs and symptoms at such an early age. Regarding the SAQ, as a recent study suggests, adolescents may have difficulties in understanding the questions and drawings used in the SAQ questionnaire [75].

#### Height

SAQ waist scores were affected by height, such so that taller patients had better scores. In our sample, taller patients had smaller curves on average compared to others, which could have positively influenced the waist scores.

#### Self-efficacy

The worst SAQ waist scores were observed among patients who had better self-efficacy scores especially those who scored ≥35 (out of 40). Perhaps, patients, whose waist is misaligned, due to a pelvic displacement were more eager and felt more confident to succeed with the treatment, thus having higher SEQ scores. A high SEQ with more severe perceived waist deformity could result because waist misalignment is one of the most observable deformities by patients themselves, compared to, for example the rib hump.

### Strength and weaknesses

This RCT addressed many aspects of scientific rigor. The assessors and the statistician were blinded to the treatment allocation. As in most clinical trials involving exercises, the therapists and the patients could not be blinded. The compliance was monitored using patient/parent/therapist logbooks, which is novel in exercise studies for scoliosis. The completers’ and the overall compliance rates were high. The attrition rate was low (12 %), suggesting the current study protocol is feasible. We made efforts to standardize the treatment by developing the classification and exercise prescription algorithms. None of our participants reported using co-interventions.

There are also limitations. The SRS-22r is the most frequently used questionnaire assessing the QOL in patients with AIS after a treatment. The SAQ is increasingly used in the same population. Our results suggested that, due to a high ceiling effects, and rate of scores close to the best values, in both questionnaires, perhaps different QOL tools should be used in patients with AIS treated conservatively. Moreover, receiving additional treatment with a therapist could positively influence these self-reported outcomes by patients not blinded to the intervention received creating the possibility of an attention bias [76]. To prevent this bias, a placebo treatment group with patients receiving an equal amount of therapist attention would be required. While not actively effective on the outcomes, the placebo treatment would have to appear convincingly effective enough for the patients to remain successfully blinded. Even if such a placebo exercise treatment were available, blinding of the therapists would still not be possible. These issues are common shortfalls of scoliosis exercises studies and no prior studies have been able to use a placebo exercise treatment [29, 33–35]. Pain and self-image are subjective outcomes, and having measured them on the subjective scales, such as SRS-22r and SAQ was an appropriate strategy. To further control the possibility of bias of subjective measurements by the absence of participants’ blinding, a battery of objective outcomes was also collected in addition to the Sorensen test. Cobb angles and posture measurements were assessed using objective quantitative procedures by blinded observers and will be reported in future publications.

Further, the study design, does not allow determining if exercises could replace bracing. To answer such a question, our study would have to randomize patients meeting the brace prescription criteria into an exercise only or a brace only group. Not offering a brace treatment to the patients meeting criteria is an ethical concern [22]. The primary goal was to determine the effect of the Schroth exercises as an add-on to the standard of care, and not as a stand-alone therapy.

## Conclusion

In summary, Schroth exercises in conjunction with the standard of care improved pain, self-image and back muscle endurance in patients with AIS over a 6-month long intervention. Other outcomes did not differ significantly between groups. The study demonstrated a high prevalence of ceiling effects and best scores on both questionnaires. There seems to be the need for using more responsive questionnaires to capture changes in conservatively treated patients with AIS and with curves ≤45 °. QOL is an important outcome [52], but does not correlate well with the curve magnitude [77], especially when curves are smaller [78]. Hence, it should be routinely tested in studies that assess the effectiveness of a conservative treatment in patients with AIS, which also allows for cross-study comparisons.

### Consent

Informed assent from the patients and informed parental consent for the participation in the study was obtained prior to the enrolment.

## Additional files

Additional file 1:
**Consort flow chart.** (DOCX 158 kb) Additional file 2:
**Exercise prescription for each curve type.** (DOCX 1383 kb) Additional file 3:
**SOSORT Management for bracing guidelines checklist.**(DOC 55 kb) Additional file 4:
**Adjusted mean estimates and standard errors for the SRS-22r, Spinal Appearance Questionnaire scores and the Biering-Sorensen test by visit and group predicted by the linear mixed effects models for the intention-to-treat (ITT) and per protocol (PP) analyses.** (DOCX 180 kb)

## Abbreviations

AIS: Adolescent idiopathic scoliosis
RCT: Randomized controlled trial
QOL: Quality of life
BME: Back muscle endurance
SRS-22r: Scoliosis research society 22r questionnaire
SAQ: Spinal appearance questionnaire
ITT: Intention-to-treat analysis
PP: Per protocol analysis
SEM: Standard error of measurement
AIC: Akaike information criterion
SEQ: Self-efficacy questionnaire
