# Supplementary material for: The effect of Schroth exercises added to the standard of care on the quality of life and muscle endurance in adolescents with idiopathic scoliosis—an assessor and statistician blinded randomized controlled trial: “SOSORT 2015 Award Winner”
Source: Scoliosis. 2015 Sep 18;10:24. doi: 10.1186/s13013-015-0048-5 (PMC4582716; doi:10.1186/s13013-015-0048-5)
Supplement: Additional file 1: — Consort flow chart. (DOCX 158 kb) [file 13013_2015_48_MOESM1_ESM.docx]

**Appendix 1. Consort flow chart**


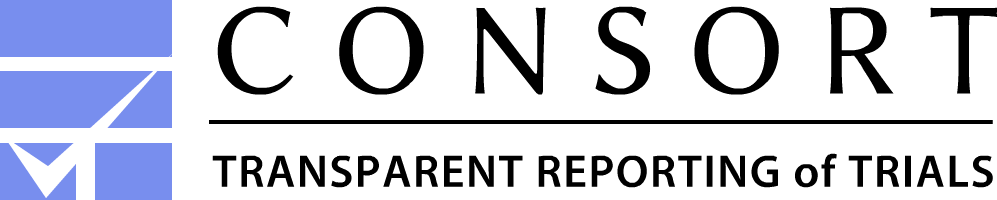


**CONSORT 2010 Flow Diagram**

## Follow-Up

Assessed for eligibility (n=2291)

## Analysis

## Enrollment

## Allocation

Randomized (n= 50)

In intention-to-treat analysed (n= 25)

In per protocol analysed (n= 21)
♦ Excluded from the per protocol analysis (n=4), only completers were considered

Lost to follow-up (n=2):

- 1 relocated

- 1 travelled for >3 months during the trial

Discontinued intervention (n= 0)

Lost to follow-up (n= 4):

- 4 reported too much time constraint

Discontinued intervention (n= 0)

Allocated to intervention (n=25)

♦ Received allocated intervention (n= 25)

♦ Did not receive allocated intervention (n= 0)

Allocated to control (n= 25)

♦ Received allocated intervention (n= 25)

♦ Did not receive allocated intervention (n= 0)

Excluded (n=2241)

♦  Not meeting inclusion criteria (n=1799)

♦  Declined to participate due to lack of interest (n=442)

♦  Other reasons (n=0)

In intention-to-treat analysed (n= 25)

In per protocol analysed (n= 23)
♦ Excluded from the per protocol analysis (n=2), only completers were considered
