# Supplementary material for: The effect of Schroth exercises added to the standard of care on the quality of life and muscle endurance in adolescents with idiopathic scoliosis—an assessor and statistician blinded randomized controlled trial: “SOSORT 2015 Award Winner”
Source: Scoliosis. 2015 Sep 18;10:24. doi: 10.1186/s13013-015-0048-5 (PMC4582716; doi:10.1186/s13013-015-0048-5)
Supplement: Additional file 2: — Exercise prescription for each curve type. (DOCX 1383 kb) [file 13013_2015_48_MOESM2_ESM.docx]

**Appendix 2. Exercise prescription for each curve type**
