# Supplementary material for: The effect of Schroth exercises added to the standard of care on the quality of life and muscle endurance in adolescents with idiopathic scoliosis—an assessor and statistician blinded randomized controlled trial: “SOSORT 2015 Award Winner”
Source: Scoliosis. 2015 Sep 18;10:24. doi: 10.1186/s13013-015-0048-5 (PMC4582716; doi:10.1186/s13013-015-0048-5)
Supplement: Additional file 4: — Adjusted mean estimates and standard errors for the SRS-22r, Spinal Appearance Questionnaire scores and the Biering-Sorensen test by visit and group predicted by the linear mixed effects models for the intention-to-treat (ITT) and per protocol (PP) analyses. (DOCX 180 kb) [file 13013_2015_48_MOESM4_ESM.docx]

**Appendix 3.** Adjusted mean estimates and standard errors for the SRS-22r, Spinal Appearance Questionnaire scores and the Biering-Sorensen test by visit and group predicted by the linear mixed effects models for the intention-to-treat (ITT) and per protocol (PP) analyses.

**Linear mixed effects model coefficients with associated standard errors (SE) and significance estimates in the intention-to-treat (ITT) and the per protocol (PP) analyses for SRS-22 function, pain, self-image and total domains.**

|  | | **ITT (N=50)** | | | | **PP (N=44)** | | | |
| --- | --- | --- | --- | --- | --- | --- | --- | --- | --- |
|  | | *Value* | *SE* | *DF* | *p-value* | *Value* | *SE* | *DF* | *p-value* |
| **(SRS-22r function)^4^** | | | | | | | | | |
| Interaction group * time3 | | 15.93 | 36.80 | 84 | 0.67 | 4.59 | 38.40 | 81 | 0.90 |
| Interaction group * time2 | | 30.05 | 38.02 | 84 | 0.43 | 33.23 | 38.43 | 81 | 0.39 |
| Group | | 30.98 | 36.12 | 44 | 0.40 | 18.00 | 35.98 | 39 | 0.62 |
| Time3 | | -17.62 | 26.22 | 84 | 0.50 | -19.58 | 26.99 | 81 | 0.47 |
| Time2 | | 12.40 | 26.77 | 81 | 0.39 | 10.36 | 27.027 | 81 | 0.70 |
| ***Covariates*:** | |  | | | | | | | |
| Weight | | 3.23 | 1.84 | 44 | 0.09 | / | / | / | / |
| Classification 3cp | | -127.18 | 46.51 | 44 | 0.01 | -161.60 | 48.58 | 39 | 0.00 |
| Classification 4c | | -126.95 | 59.95 | 44 | 0.04 | 4.59 | 38.40 | 81 | 0.90 |
| Classification 4cp | | -107.61 | 44.29 | 44 | 0.02 | 33.23 | 38.43 | 81 | 0.39 |
| **(SRS-22r pain)^4^** | | | | | | | | | |
| Interaction group * time3 | | -29.16 | 38.39 | 84 | 0.45 | -33.17 | 39.61 | 80 | 0.40 |
| Interaction group * time2 | | 85.25 | 39.386 | 84 | 0.02 | 81.54 | 39.42 | 80 | 0.04 |
| Group | | 74.21 | 45.82 | 47 | 0.11 | 95.22 | 46.09 | 38 | 0.05 |
| Time3 | | 67.08 | 27.11 | 84 | 0.01 | 61.19 | 27.64 | 80 | 0.03 |
| Time2 | | -20.02 | 27.38 | 84 | 0.47 | -17.33 | 27.58 | 80 | 0.53 |
| ***Covariates*:** | |  | | | | | | | |
| Age | | -20.20 | 12.25 | 47 | 0.11 | -23.71 | 12.62 | 38 | 0.07 |
| Self-efficacy | | 4.82 | 2.94 | 84 | 0.10 | 4.84 | 2.97 | 80 | 0.11 |
| **SRS-22r self-image** | | | | | | | | | |
| Interaction group * time3 | | -0.22 | 0.15 | 85 | 0.14 | -0.18 | 0.15 | 82 | 0.25 |
| Interaction group * time2 | | 0.30 | 0.15 | 85 | 0.05 | 0.29 | 0.15 | 82 | 0.06 |
| Group | | 0.09 | 0.16 | 47 | 0.57 | 0.12 | 0.17 | 38 | 0.51 |
| Time3 | | 0.06 | 0.10 | 85 | 0.53 | 0.05 | 0.11 | 82 | 0.62 |
| Time2 | | -0.17 | 0.10 | 85 | 0.11 | -0.17 | 0.11 | 82 | 0.12 |
| ***Covariates*:** | |  | | | | | | | |
| Brace wear | | 0.28 | 0.15 | 47 | 0.07 | 0.27 | 0.15 | 38 | 0.09 |
| **SRS-22r total** | | | | | | | | | |
| Interaction group * time3 | | 0.02 | 0.08 | 82 | 0.83 | 0.02 | 0.08 | 80 | 0.82 |
| Interaction group * time2 | | 0.14 | 0.08 | 82 | 0.08 | 0.12 | 0.08 | 80 | 0.11 |
| Group | | 0.10 | 0.10 | 46 | 0.35 | 0.14 | 0.11 | 39 | 0.19 |
| Time3 | | 0.03 | 0.05 | 82 | 0.59 | 0.01 | 0.05 | 80 | 0.77 |
| Time2 | | -0.03 | 0.05 | 82 | 0.58 | -0.02 | 0.05 | 80 | 0.63 |
| ***Covariates*:** | |  | | | | | | | |
| ***ITT*** | ***PP*** |  |  |  |  |  |  |  |  |
| Age | Class.3cp | -0.08 | 0.04 | 46 | 0.05 | -0.42 | 0.15 | 39 | 0.01 |
| Height | Class.4c | 1.06 | 0.74 | 46 | 0.16 | -0.29 | 0.19 | 39 | 0.14 |
| SEQ | Class.4cp | 0.01 | 0.01 | 82 | 0.16 | -0.48 | 0.15 | 39. | 0.00 |

**Legend:** *Interaction group*time3* represents the effect of the treatment after 3 months; *Interaction group*time2* represents the effect of the treatment after 6 months; *Group* main effect of group, *time* main effect of time, *time3* main effect of the follow-ups, *weight* main effect of weight, *Classification 3cp* main effect of the 3cp classification, *Classification 4c* main effect of the 4c classification, *Classification 4cp* main effect of the 4cp classification, *age* main effect of age, *brace wear* main effect of the brace-wear.

**Linear mixed effects model coefficients with associated standard errors (SE) and significance estimates in the intention-to-treat (ITT) and the per protocol (PP) analyses for the SAQ domains.**

|  | | **ITT (N=50)** | | | | **PP (N=44)** | | | |
| --- | --- | --- | --- | --- | --- | --- | --- | --- | --- |
|  | | *Value* | *SE* | *DF* | *p-value* | *Value* | *SE* | *DF* | *p-value* |
| **SAQ general** | | | | | | | | | |
| Interaction group * time3 | | 0.08 | 0.22 | 86 | 0.71 | -0.00 | 0.23 | 83 | 0.98 |
| Interaction group * time2 | | -0.12 | 0.23 | 86 | 0.60 | -0.14 | 0.23 | 83 | 0.56 |
| Group | | 0.04 | 0.24 | 46 | 0.88 | 0.23 | 0.27 | 36 | 0.39 |
| Time3 | | -0.02 | 0.16 | 86 | 0.89 | 0.00 | 0.16 | 83 | 0.97 |
| Time2 | | 0.09 | 0.16 | 86 | 0.58 | 0.11 | 0.16 | 83 | 0.51 |
| ***Covariates*:** | |  | | | | | | | |
| ***ITT*** | ***PP*** |  | | | | | | | |
| Height | Height | 2.15 | 1.27 | 46 | 0.10 | 6.45 | 1.99 | 36 | 0.00 |
| Brace wear | Brace wear | -0.72 | 0.23 | 46 | 0.00 | -1.10 | 0.27 | 36 | 0.00 |
| / | Age | / | / | / | / | -0.27 | 0.12 | 36 | 0.02 |
| / | Class.3cp | / | / | / | / | 1.09 | 0.40 | 36 | 0.01 |
| / | Class.4c | / | / | / | / | 0.77 | 0.45 | 36 | 0.09 |
| / | Class.4cp | / | / | / | / | 0.86 | 0.35 | 36 | 0.02 |
| **(SAQ waist)^-0.3^** | | | | | | | | | |
| Interaction group * time3 | | -0.03 | 0.04 | 83 | 0.46 | -0.00 | 0.04 | 80 | 0.91 |
| Interaction group * time2 | | -0.02 | 0.04 | 83 | 0.58 | -0.02 | 0.04 | 80 | 0.60 |
| Group | | -0.01 | 0.04 | 47 | 0.88 | -0.03 | 0.05 | 41 | 0.46 |
| Time3 | | 0.00 | 0.03 | 83 | 0.88 | -0.00 | 0.03 | 80 | 0.85 |
| Time2 | | -0.01 | 0.03 | 83 | 0.80 | -0.01 | 0.03 | 80 | 0.73 |
| ***Covariates*:** | |  | | | | | | | |
| SEQ | | 0.01 | 0.00 | 83 | 0.09 | 0.01 | 0.00 | 80 | 0.16 |
| SEQ 2 | | 0.10 | 0.04 | 83 | 0.01 | 0.09 | 0.04 | 80 | 0.02 |
| Brace wear | | 0.08 | 0.04 | 47 | 0.03 | 0.09 | 0.04 | 41 | 0.03 |
| **SAQ shoulder** | | | | | | | | | |
| Interaction group * time3 | | -0.23 | 0.26 | 85 | 0.38 | 0.12 | 0.25 | 83 | 0.62 |
| Interaction group * time2 | | 0.32 | 0.25 | 85 | 0.21 | -0.26 | 0.25 | 83 | 0.30 |
| Group | | -0.09 | 0.29 | 48 | 0.76 | 0.24 | 0.29 | 42 | 0.40 |
| Time3 | | -0.06 | 0.17 | 85 | 0.75 | -0.01 | 0.18 | 83 | 0.97 |
| Time2 | | 0.18 | 0.18 | 85 | 0.30 | 0.19 | 0.18 | 83 | 0.28 |
| **SAQ trunk shift** | | | | | | | | | |
| Interaction group * time3 | | 0.23 | 0.22 | 85 | 0.30 | 0.13 | 0.23 | 83 | 0.58 |
| Interaction group * time2 | | -0.25 | 0.22 | 85 | 0.26 | -0.26 | 0.23 | 83 | 0.26 |
| Group | | 0.01 | 0.16 | 43 | 0.97 | 0.11 | 0.18 | 37 | 0.53 |
| Time3 | | -0.05 | 0.15 | 85 | 0.75 | -0.03 | 0.16 | 83 | 0.84 |
| Time2 | | 0.24 | 0.15 | 85 | 0.12 | 0.26 | 0.16 | 83 | 0.10 |
| ***Covariates*:** | |  | | | | | | | |
| Age 10-11 | | -0.83 | 0.25 | 43 | 0.00 | -0.87 | 0.26 | 37 | 0.00 |
| Height | | -1.89 | 0.76 | 43 | 0.02 | -1.43 | 0.77 | 37 | 0.07 |
| Classification 3cp | | 0.49 | 0.19 | 43 | 0.01 | 0.55 | 0.189 | 37 | 0.01 |
| Classification 4c | | 0.10 | 0.23 | 43 | 0.67 | 0.12 | 0.23 | 37 | 0.60 |
| Classification 4cp | | 0.36 | 0.17 | 43 | 0.05 | 0.40 | 0.18 | 37 | 0.03 |
| **log (SAQ chest)** | | | | | | | | | |
| Interaction group * time3 | | -0.00 | 0.18 | 85 | 0.99 | -0.01 | 0.19 | 82 | 0.95 |
| Interaction group * time2 | | 0.22 | 0.14 | 85 | 0.13 | 0.23 | 0.15 | 82 | 0.14 |
| Group | | 0.10 | 0.16 | 42 | 0.53 | -0.06 | 0.17 | 36 | 0.73 |
| Time3 | | 0.19 | 0.13 | 85 | 0.14 | 0.18 | 0.13 | 82 | 0.17 |
| Time2 | | -0.06 | 0.10 | 85 | 0.56 | -0.07 | 0.11 | 82 | 0.51 |
| ***Covariates*:** | |  | | | | | | | |
| I Age | | -0.06 | 0.02 | 42 | 0.01 | -0.06 | 0.02 | 36 | 0.01 |
| Age 13 | | -0.48 | 0.18 | 42 | 0.01 | -0.50 | 0.20 | 36 | 0.02 |
| Brace wear | | -0.38 | 0.14 | 42 | 0.01 | -0.44 | 0.15 | 36 | 0.01 |
| Classification 3cp | | 0.42 | 0.21 | 42 | 0.06 | 0.47 | 0.23 | 36 | 0.05 |
| Classification 4c | | 0.65 | 0.27 | 42 | 0.02 | 0.67 | 0.29 | 36 | 0.02 |
| Classification 4cp | | 0.51 | 0.21 | 42 | 0.02 | 0.57 | 0.23 | 36 | 0.02 |
| $\sqrt{\boldsymbol{SAQ prominence}}$ | | | | | | | | | |
| Interaction group * time3 | | 0.13 | 0.07 | 85 | 0.06 | 0.12 | 0.07 | 83 | 0.10 |
| Interaction group * time2 | | -0.07 | 0.07 | 85 | 0.33 | -0.08 | 0.07 | 83 | 0.28 |
| Group | | -0.02 | 0.06 | 45 | 0.67 | 0.02 | 0.06 | 38 | 0.79 |
| Time3 | | -0.00 | 0.04 | 85 | 0.98 | 0.01 | 0.05 | 83 | 0.78 |
| Time2 | | 0.06 | 0.05 | 85 | 0.20 | 0.07 | 0.05 | 83 | 0.19 |
| ***Covariates***: | |  | | | | | | | |
| ***ITT*** | ***PP*** |  | | | | | | | |
| Class.3cp | Class.3cp | 0.25 | 0.08 | 45 | 0.00 | 0.21 | 0.079 | 38 | 0.01 |
| Class.4c | Class.4c | 0.01 | 0.10 | 45 | 0.92 | -0.02 | 0.10 | 38 | 0.86 |
| Class.4cp | Class.4cp | 0.14 | 0.07 | 45 | 0.06 | 0.09 | 0.07 | 38 | 0.23 |
|  | Brace wear |  |  |  |  | -0.09 | 0.05 | 38 | 0.08 |

**Legend:** *Interaction group*time3* represents the effect of the treatment after 3 months; *Interaction group*time2* represents the effect of the treatment after 6 months; *Group* main effect of group, *time* main effect of time, *time3* main effect of the follow-ups, *height* main effect of height, *brace wear* main effect of the brace-wear, *SEQ* main effect of SEQ score, *SEQ2* main effect of the SEQ scores when ≥35, *Age 10-11* main effect of the age 10 and 11, *Classification 3cp* main effect of the 3cp classification, *Classification 4c* main effect of the 4c classification, *Classification 4cp* main effect of the 4cp classification, *age* main effect of age, *I age* main effect of the quadratic function of age, *Age 13* main effect of age 13.

**Linear mixed effects model coefficients with associated standard errors (SE) and significance estimates in the intention-to-treat (ITT) and the per protocol (PP) analyses for the Biering-Sorensen test.**

|  | **ITT (N=50)** | | | | **PP (N=44)** | | | |
| --- | --- | --- | --- | --- | --- | --- | --- | --- |
| **Biering-Sorensen test** | *Value* | *SE* | *DF* | *p-value* | *Value* | *SE* | *DF* | *p-value* |
| Interaction group * time3 | 27.5 | 13.43 | *84* | *0.04* | 25.9 | 13.41 | *81* | *0.05* |
| Interaction group * time2 | -1.9 | 13.55 | 84 | 0.89 | 2.3 | 13.30 | 81 | 0.86 |
| Group | -3.6 | 16.31 | *46* | *0.83* | -5.6 | 18.00 | *40* | *0.76* |
| Time3 | 4.8 | 9.56 | *84* | *0.62* | 3.4 | 9.30 | *81* | *0.71* |
| Time2 | 6.3 | 9.51 | *84* | *0.54* | 5.5 | 9.21 | *81* | *0.55* |
| ***Covariates*:** |  | | | | | | | |
| Age | 9.5 | 4.75 | *46* | *0.05* | 9.5 | 5.26 | *40* | *0.08* |
| SEQ | -1.6 | 1.06 | *84* | *0.15* | -1.6 | 1.07 | *81* | *0.13* |
| Brace wear | 25.0 | 16.86 | *46* | *0.14* | 28.0 | 18.48 | *40* | *0.14* |

**Legend:** *Interaction group*time3* represents the effect of the treatment after 3 months; *Interaction group*time2* represents the effect of the treatment after 6 months; *Group* main effect of group, *time* main effect of time, *time3* main effect of the follow-ups, *age* main effect of age, *SEQ* main effect of the SEQ score, *brace wear* main effect of the brace-wear.
